# Nationwide survey of the development of drug resistance in the pediatric field in 2007 and 2010: drug sensitivity of *Haemophilus influenzae* in Japan (second report)

**DOI:** 10.1007/s10156-013-0591-z

**Published:** 2013-04-07

**Authors:** Tadashi Hoshino, Yoshitake Sato, Yoshikiyo Toyonaga, Hideaki Hanaki, Keisuke Sunakawa, The Drug-Resistant Pathogen Surveillance Group in Pediatric Infectious Disease

**Affiliations:** 1 Division of Infectious Diseases, Chiba Children’s Hospital, 579-1 Heta-cho, Midori-ku, Chiba, Chiba Japan; 2 Department of Pediatrics, Fiji Heavy Industries Health Insurance Society Ota Memorial Hospital, Ota, Japan; 3 Department of Pediatrics, Sekishinkai Sayama Hospital, Sayama, Japan; 4 The Kitasato Institute, Kitasato University Research Center for Anti-infectious Drugs, Tokyo, Japan; 5 The Kitasato Institute, Kitasato University Research Organization for Infection Control Sciences, Tokyo, Japan

**Keywords:** Pediatric infectious disease, Surveillance, *Haemophilus influenzae* sensitivity, Drug resistance

## Abstract

The Drug-Resistant Pathogen Surveillance Group in Pediatric Infectious Disease conducted national surveillance for *Haemophilus influenzae* in 2007 (phase 3) and 2010 (phase 4), following the previous surveillance conducted from 2000 to 2001 (phase 1) and in 2004 (phase 2). We examined the antimicrobial susceptibility for *H. influenzae* derived from clinical specimens of pediatric patients collected nationwide from 27 institutions during phases 3 (386 strains) and 4 (484 strains). The frequency of β-lactamase-nonproducing ampicillin (ABPC)-resistant (BLNAR) strains, which rapidly increased from 11.4 % in phase 1 to 43.4 % in phase 2, has gradually decreased from 38.3 % in phase 3 to 37.8 % in phase 4. In contrast, On the other hand, the frequency of β-lactamase-producing strains, which continuously decreased from 8.3 % in phase 1 to 4.4 % in phase 3, has increased to 8.7 % in phase 4. Prevalence of β-lactamase-producing clavulanic acid/amoxicillin-resistant (BLPACR) strains, especially, has increased from 1.6 % in phase 3 to 4.8 % in phase 4. The oral antimicrobial agents with the lowest MIC_90_ were levofloxacin in both phases, and tosufloxacin in phase 4 (≤0.063 μg/ml), whereas for intravenous use the corresponding agent was tazobactam/piperacillin in both phases (0.125 μg/ml). There was no increase in the MIC_90_ of most β-lactams between phase 3 and phase 4. In relationship to sex, age, presence of siblings, attendance at a daycare center, siblings’ attendance at a daycare center, and prior administration of antimicrobial agents within 1 month, the frequency of β-lactamase-nonproducing ABPC-intermediately resistant (BLNAI) strains + BLNAR strains was high (*P* = 0.005) in cases with prior administration of antimicrobial agents in phase 3.

## Introduction

*Haemophilus influenzae*, along with *Streptococcus pneumoniae*, is a major pathogen in respiratory tract infection and invasive infection in children. Previously, *H. influenzae* developed resistance to ampicillin (ABPC) by producing β-lactamase; however, since the beginning of the 2000s, there has been a rapid increase in the prevalence of ampicillin-resistant strains that do not produce β-lactamase, that is, β-lactamase-nonproducing ABPC-resistant (BLNAR) strains [1]. With regard to the BLNAR strains, their sensitivity to cephems and carbapenems, as well as to ABPC, decreases as a result of mutations in the *ftsI* genes that encode penicillin-binding protein (PBP) 3 [2], causing major problems in the development of treatment strategies for pediatric infection such as meningitis. The Drug-Resistant Pathogen Surveillance Group in Pediatric Infectious Disease reported that from phase 1 (2000–2001) to phase 2 (2004) of Nationwide Surveillance there was a rapid increase in the distribution of BLNAR strains and a decrease in β-lactamase-nonproducing ABPC-sensitive (BLNAS) strains [3]. It is very important to maintain an understanding of the trends in development of drug resistance in *H. influenzae* to be able to choose the proper antimicrobial agent in situations where there are significant changes in the prevalence of drug-resistant strains. We therefore conducted phase 3 (2007) and phase 4 (2010) surveillance studies, following the first two phases. Here we report the results of these studies.

## Materials and methods

### Strains, antimicrobial susceptibility testing, and capsular typing for serotype b strains

We collected *H. influenzae* isolated from clinical specimens taken from pediatric patients at 27 institutions nationwide, all of which participated in the Drug-Resistant Pathogen Surveillance Group in Pediatric Infectious Disease, and used the 386 strains accumulated from January to June in 2007 for phase 3 and the 484 strains accumulated from January to June in 2010 for phase 4. The sources of the isolates were as follows in phase 3: nasopharynx, 299 strains; pharynx, 51 strains; sputum, 29 strains; blood, 3 strains; nasal discharge and pus, 1 strain for each; and unknown origin, 2 strains. Sources of isolates were as follows in phase 4: nasopharynx, 396 strains; sputum, 40 strains; pharynx, 30 strains; blood, 8 strains; cerebrospinal fluid, 4 strains; and unknown origin, 6 strains.

For antimicrobial susceptibility testing, we measured the minimum inhibitory concentration (MIC) by the broth microdilution method, complying with the Clinical and Laboratory Standards Institute (CLSI) standards [4]. Sensitivity to the following 21 drugs was tested during phase 3: ABPC, clavulanic acid/amoxicillin (CVA/AMPC), piperacillin (PIPC), tazobactam/piperacillin (TAZ/PIPC), cefaclor (CCL), cefditoren (CDTR), cefcapene (CFPN), cefpodoxime (CPDX), cefdinir (CFDN), cefotaxime (CTX), cefteram (CFTM), cefotiam (CTM), ceftriaxone (CTRX), faropenem (FRPM), panipenem (PAPM), meropenem (MEPM), azithromycin (AZM), clarithromycin (CAM), rokitamycin (RKM), telithromycin (TEL), and levofloxacin (LVFX). During phase 4, sensitivity was tested against a total of 23 drugs including those tested during phase 3 (other than TEL), with the following additional drugs: tebipenem (TBPM), doripenem (DRPM), and tosufloxacin (TFLX). β-Lactamase production was determined by the nitrocephin method.

The strains were classified according to the CLSI criteria [5]: that is, β-lactamase-nonproducing strains were classified into BLNAS strains, for which the MIC for ABPC was 1 μg/ml or less; β-lactamase-nonproducing ABPC-intermediately resistant (BLNAI) strains, with MIC for ABPC of 2 μg/ml; and BLNAR strains, with MIC for ABPC of 4 μg/ml or more. β-Lactamase-producing strains were classified into β-lactamase-producing ABPC-resistant (BLPAR) strains, with MIC for CVA/AMPC of 4 μg/ml or less; and β-lactamase-producing CVA/AMPC-resistant (BLPACR) strains, with MIC for CVA/AMPC of 8 μg/ml or more.

Additionally, all the strains were tested for *H. influenzae* serotype b (Hib) by the polymerase chain reaction (PCR) method [6]. Fourteen strains (3.6 %) in phase 3 and 23 strains (4.8 %) in phase 4 were detected as Hib. The sources of Hib strains in phase 3 were as follows: nasopharynx, 9 strains; blood, 3 strains; and pharynx and pus, 1 strain for each. Sources of Hib strains in phase 4: nasopharynx, 9 strains; blood, 8 strains; cerebrospinal fluid, 4 strains; and sputum and pharynx, 1 strain each.

### Background factors and statistical analysis

To examine the relationship between background factors and the development of drug resistance, the frequency of isolation was compared between drug-resistant strains and BLNAS strains in relationship to six background factors, including sex, age, presence or absence of siblings, attendance or nonattendance at a daycare center, siblings’ attendance or nonattendance at a daycare center, and prior administration of antimicrobial agents within 1 month. The standard for drug-resistant strains was the same as that used in phase 1 and phase 2 and was defined as BLNAI + BLNAR. The χ^2^ test was used to identify whether a significant difference exists, using two-sided testing at a 5 % level of significance. Fisher’s exact test was used when an expected value was less than 5.

## Results

**Fig. 1:**
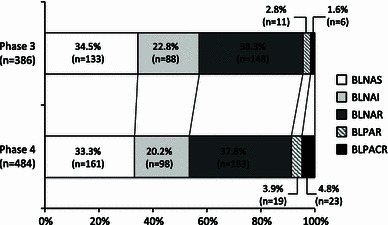
Distribution of *Haemophilus influenzae* strains classified by ampicillin (ABPC) or clavulanic acid/amoxicillin (CVA/AMPC) resistance in phases 3 and 4. *BLNAS*, β-lactamase-nonproducing ABPC-sensitive strain; *BLNAI*, β-lactamase-nonproducing ABPC-intermediately resistant strain; *BLNAR*, β-lactamase-nonproducing ABPC-resistant strain; *BLPAR*, β-lactamase-producing ABPC-resistant strain; *BLPACR*, β-lactamase-producing CVA/AMPC-resistant strain

Figure 1 shows the number of strains by degrees of resistance. In phase 3, there were 133 strains of BLNAS (34.5 %), 88 strains of BLNAI (22.8 %), 148 strains of BLNAR (38.3 %), 11 strains of BLPAR (2.8 %), and 6 strains of BLPACR (1.6 %). In phase 4, there were 161 strains of BLNAS (33.3 %), 98 strains of BLNAI (20.2 %), 183 strains of BLNAR (37.8 %), 19 strains of BLPAR (3.9 %), and 23 strains of BLPACR (4.8 %).

**Fig. 2:**
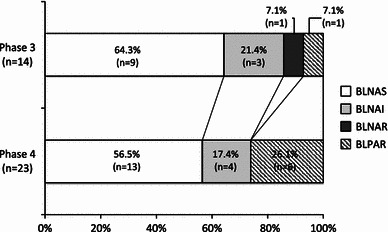
Distribution of *Haemophilus influenzae* serotype b strains classified by ampicillin (ABPC) or clavulanic acid/amoxicillin (CVA/AMPC) resistance in phases 3 and 4. *BLNAS*, β-lactamase-nonproducing ABPC-sensitive strain; *BLNAI*, β-lactamase-nonproducing ABPC-intermediately resistant strain; *BLNAR*, β-lactamase-nonproducing ABPC-resistant strain; *BLPAR*, β-lactamase-producing ABPC-resistant strain; *BLPACR*, β-lactamase-producing CVA/AMPC-resistant strain

Figure 2 shows the number of Hib strains by degrees of resistance. In phase 3, there were 9 strains of BLNAS (64.3 %), 3 strains of BLNAI (21.4 %), 1 strain of BLNAR (7.1 %), 1 strain of BLPAR (7.1 %), and no BLPACR strain. In phase 4, there were 13 strains of BLNAS (56.5 %), 4 strains of BLNAI (17.4 %), 6 strains of BLPAR (26.1 %), and no BLNAR or BLPACR strains.

Table 1 shows the MIC_50_, MIC_90_, and MIC range of antimicrobial agents for *H. influenzae*. In phase 3 with a total of 386 strains, the oral antimicrobial agent with the lowest MIC_90_ was LVFX (≤0.063 μg/ml), followed by CDTR (0.25 μg/ml). The intravenous antimicrobial agent with the lowest MIC_90_ was TAZ/PIPC (0.125 μg/ml), followed by PIPC, CTRX, and MEPM (0.25 μg/ml). In phase 4 with a total of 484 strains, the oral antimicrobial agent with the lowest MIC_90_ was LVFX and TFLX (≤0.063 μg/ml), followed by CDTR (0.25 μg/ml). The intravenous antimicrobial agent with the lowest MIC_90_ was TAZ/PIPC (0.125 μg/ml), followed by PIPC and CTRX (0.25 μg/ml). Between phase 3 and phase 4, there was a twofold increase in the MIC_90_ values of PAPM (2–4 μg/ml) and MEPM (0.25–0.5 μg/ml), but there were no changes in those of other drugs.

**Table 1:** Susceptibilities for *Haemophilus influenzae* in phase 3 and phase 4

	Phase 3			Phase 4		
Number of strains	386			484		
MIC	MIC_50_	MIC_90_	MIC range	MIC_50_	MIC_90_	MIC range
ABPC	2	8	0.12–>128	2	8	≤0.063–>128
CVA/AMPC	4	8	0.25–32	4	8	0.125–16
PIPC	≤0.063	0.25	≤0.063–>128	≤0.063	0.25	≤0.063–>128
TAZ/PIPC	≤0.063	0.125	≤0.063–1	≤0.063	0.125	≤0.063–0.25
CCL	16	64	0.25–>128	16	64	0.25–128
CDTR	0.125	0.25	≤0.063–0.5	0.125	0.25	≤0.063–1
CFPN	1	2	≤0.063–4	1	2	≤0.063–8
CPDX	2	4	≤0.063–8	2	4	≤0.063–8
CFDN	2	8	≤0.063–16	2	8	≤0.063–16
CFTM	0.5	1	≤0.063–2	0.5	1	≤0.063–2
CTM	8	64	≤0.063–128	8	64	0.125–128
CTRX	0.125	0.25	≤0.063–0.5	0.125	0.25	≤0.063–0.5
CTX	0.5	1	≤0.063–4	0.5	1	≤0.063–4
AZM	1	2	0.125–64	1	2	≤0.063–4
CAM	4	8	1–>64	4	8	0.5–32
RKM	8	16	1–32	8	16	≤0.063–32
TEL	1	2	0.25–8	–	–	–
FRPM	2	4	≤0.063–4	2	4	≤0.063–8
TBPM	–	–	–	0.25	1	≤0.063–2
PAPM	1	2	≤0.063–4	1	4	≤0.063–8
MEPM	0.125	0.25	≤0.063–1	0.125	0.5	≤0.063–1
DRPM	–	–	–	0.5	2	≤0.063–4
LVFX	≤0.063	≤0.063	≤0.063–0.5	≤0.063	≤0.063	≤0.063–0.5
TFLX	–	–	–	≤0.063	≤0.063	≤0.063–2

*ABPC* ampicillin, *CVA/AMPC* clavulanic acid/amoxicillin, *PIPC* piperacillin, *TAZ/PIPC* tazobactam/piperacillin, *CCL* cefaclor, *CDTR* cefditoren, *CFPN* cefcapene, *CPDX* cefpodoxime, *CFDN* cefdinir, *CFTM* cefteram, *CTM* cefotiam, *CTRX* ceftriaxone, *CTX* cefotaxime, *AZM* azithromycin, *CAM* clarithromycin, *RKM* rokitamycin, *TEL* telithromycin, *FRPM* faropenem, *TBPM* tebipenem, *PAPM* panipenem, *MEPM* meropenem, *DRPM* doripenem, *LVFX* levofloxacin, *TFLX* tosufloxacin

Table 2 shows the MIC_50_, MIC_90_, and MIC range of antimicrobial agents for *H. influenzae* divided into the following five groups by degrees of resistance, the BLNAS, BLNAI, BLNAR, BLPAR, and BLPACR groups, respectively. In the BLNAR group, the MIC_50_ values of the β-lactams excluding PIPC and TAZ/PIPC were 4- to 64 fold higher, and the MIC_90_ values of all the agents were 2- to 4 fold higher than the values in the BLNAS group in phase 3. In phase 4, in the BLNAR group, the MIC_50_ values of the β-lactams excluding PIPC and TAZ/PIPC were 4- to 32 fold higher, and the MIC_90_ values of all the agents were 2- to 8 fold higher than the values in the BLNAS group. In both phase 3 and phase 4, the MIC_50_ values of PIPC and TAZ/PIPC were the same in the BLNAS and BLNAR groups (≤0.063 μg/ml).

**Table 2:** Suscepsibilities for *Haemophilus influenzae* (divided into five groups) in phase 3 and phase 4

Class	BLNAS						BLNAI						BLNAR					
Phase	Phase 3			Phase 4			Phase 3			Phase 4			Phase 3			Phase 4		
No. of strains (%)	133 (34.5)			161(33.3)			88 (22.8)			98 (20.2)			148 (38.3)			183 (37.8)		
MIC	MIC_50_	MIC_90_	MIC Range	MIC_50_	MIC_90_	MIC Range	MIC_50_	MIC_90_	MIC Range	MIC50	MIC90	MIC Range	MIC_50_	MIC_90_	MIC Range	MIC_50_	MIC_90_	MIC Range
ABPC	0.25	1	0.125–1	0.5	1	≤0.063–1	2	2	2–2	2	2	2–2	4	8	4–16	4	8	4–8
CVA/AMPC	0.5	2	0.25–4	0.5	2	0.125–4	4	8	1–8	4	4	1–8	8	8	2–32	4	8	2–16
PIPC	≤0.063	0.125	≤0.063–0.5	≤0.063	0.125	≤0.063–0.5	≤0.063	0.25	≤0.063–0.5	≤0.063	0.125	≤0.063–0.25	≤0.063	0.25	≤0.063–1	≤0.063	0.25	≤0.063–0.5
TAZ/PIPC	≤0.063	≤0.063	≤0.063–0.5	≤0.063	≤0.063	≤0.063–0.25	≤0.063	0.125	≤0.063–0.25	≤0.063	0.125	≤0.063–0.25	≤0.063	0.25	≤0.063–1	≤0.063	0.125	≤0.063–0.25
CCL	4	16	0.25–64	4	16	0.25–64	32	64	4–>128	16	64	2–128	64	128	4–to 128	32	64	2–128
CDTR	≤0.063	≤0.063	≤0.063–0.5	≤0.063	0.125	≤0.063–0.5	0.125	0.25	≤0.063–0.5	0.25	0.25	≤0.063–0.5	0.25	0.25	≤0.063–0.5	0.25	0.5	≤0.063–1
CFPN	≤0.063	0.5	≤0.063–2	≤0.063	0.5	≤0.063–1	1	2	≤0.063–4	1	2	≤0.063–4	2	2	0.25–4	2	4	≤0.063–8
CPDX	≤0.063	1	≤0.063–8	≤0.063	1	≤0.063–4	2	8	0.25–8	2	4	0.125–8	4	4	1–8	2	4	0.25–8
CFDN	0.25	2	≤0.063–8	0.5	2	≤0.063–4	4	8	0.5–16	2	4	0.5–8	4	8	0.5–16	4	8	0.5–16
CFTM	≤0.063	0.5	≤0.063–1	≤0.063	0.5	≤0.063–1	0.5	1	≤0.063–1	0.5	1	≤0.063–2	0.5	1	0.125–2	1	1	≤0.063–1
CTM	2	8	≤0.063–64	2	8	0.125–32	16	64	2–64	8	32	1–64	32	64	2–128	32	64	1–128
CTRX	≤0.063	0.125	≤0.063–0.25	≤0.063	0.125	≤0.063–0.25	0.125	0.25	≤0.063–0.5	0.125	0.25	≤0.063–0.5	0.25	0.25	≤0.063–0.5	0.25	0.25	≤0.063–0.5
CTX	≤0.063	0.5	≤0.063–1	≤0.063	0.5	≤0.063–0.5	0.5	1	≤0.063–2	0.5	1	≤0.063–2	0.5	2	0.125–4	1	2	≤0.063–4
AZM	1	2	0.125–4	1	1	≤0.063–4	1	2	0.25–4	1	2	0.125–4	1	2	0.25–4	1	2	0.125–2
CAM	4	8	1–16	4	8	0.5–32	4	8	1–16	4	16	1–16	8	8	1–16	8	8	2–16
RKM	8	16	1–32	4	16	≤0.063–32	8	16	2–16	8	16	1–32	8	16	2–16	8	16	0.25–16
TEL	1	2	0.25–8	–	–	–	1	2	0.5–8	–	–	–	2	2	0.5–4	–	–	–
FRPM	0.5	1	≤0.063–2	0.5	2	≤0.063–4	2	2	0.25–4	1	2	0.25–4	2	4	0.25–4	2	4	0.5–8
TBPM	–	–	–	0.125	0.25	≤0.063–1	–	–	–	0.25	1	≤0.063–1	–	–	–	0.5	1	≤0.063–2
PAPM	0.5	1	≤0.063–2	0.5	2	≤0.063–4	1	2	0.125–4	1	4	≤0.063–8	2	2	0.125–4	2	4	0.25–8
MEPM	≤0.063	0.125	≤0.063–0.125	≤0.063	0.125	≤0.063–0.5	0.125	0.25	≤0.063–0.5	0.25	0.5	≤0.063–0.5	0.25	0.5	≤0.063–1	0.25	0.5	≤0.063–1
DRPM	–	–	–	0.125	0.5	≤0.063–1	–	–	–	0.5	1	≤0.063–2	–	–	–	1	2	≤0.063–4
LVFX	≤0.063	≤0.063	≤0.063–0.125	≤0.063	≤0.063	≤0.063–0.5	≤0.063	≤0.063	≤0.063–0.125	≤0.063	≤0.063	≤0.063–0.5	≤0.063	≤0.063	≤0.063–0.5	≤0.063	≤0.063	≤0.063–0.125
TFLX	–	–	–	≤0.063	≤0.063	≤0.063–2	–	–	–	≤0.063	≤0.063	≤0.063–≤0.063	–	–	–	≤0.063	≤0.063	≤0.063–0.125

Class	BLPAR						BLPACR					
Phase	Phase 3			Phase 4			Phase 3			Phase 4		
No. of strains (%)	11 (2.8)			19 (3.9)			6 (1.6)			23 (4.8)		
MIC	MIC_50_	MIC_90_	MIC Range	MIC_50_	MIC_90_	MIC Range	MIC_50_	MIC_90_	MIC Range	MIC_50_	MIC_90_	MIC Range
ABPC	128	>128	2 to >128	32	>128	16 to >128	>128	>128	>128 to >128	>128	>128	64 to >128
CVA/AMPC	1	4	0.25–4	2	4	0.5–4	8	16	8–16	8	16	8–16
PIPC	128	>128	0.25 to >128	16	>128	2 to >128	>128	>128	>128 to >128	64	>128	8 to >128
TAZ/PIPC	≤0.063	≤0.063	≤0.063–≤0.063	≤0.063	0.125	≤0.063–0.125	≤0.063	0.125	≤0.063–0.125	≤0.063	≤0.063	≤0.063–0.125
CCL	8	32	2–64	8	32	2–32	64	128	4–128	32	64	16–128
CDTR	≤0.063	0.125	≤0.063–0.25	≤0.063	0.25	≤0.063–0.5	0.125	0.25	0.125–0.25	0.25	0.25	≤0.063–0.25
CFPN	≤0.063	1	≤0.063–2	0.5	1	≤0.063–1	2	2	0.5–2	2	2	0.5–2
CPDX	≤0.063	2	≤0.063–4	1	2	≤0.063–2	4	4	2–4	2	4	2–4
CFDN	0.25	2	0.125–4	1	4	0.25–4	4	8	2–8	4	8	2–8
CFTM	≤0.063	0.5	≤0.063–0.5	0.5	0.5	≤0.063–0.5	0.5	1	0.5–1	0.5	1	0.25–1
CTM	2	32	0.5–64	4	16	0.5–16	64	128	8–128	32	64	8–128
CTRX	≤0.063	0.25	≤0.063–0.25	≤0.063	0.25	≤0.063–0.25	0.25	0.25	0.125–0.25	0.25	0.25	≤0.063–0.5
CTX	≤0.063	0.5	≤0.063–1	0.25	0.5	≤0.063–1	1	1	0.5–1	1	1	0.25–1
AZM	1	2	0.5–64	0.5	1	0.25–1	1	64	0.5–64	1	2	0.5–4
CAM	8	8	2 to >64	4	8	2–8	4	>64	4 to >64	8	16	4–16
RKM	8	16	4–16	4	8	1–8	2	8	2–8	8	8	2–16
TEL	2	4	1–4	–	–	–	1	8	1–8	–	–	–
FRPM	0.5	2	0.125–2	0.5	2	≤0.063–4	2	2	2–2	2	4	1–4
TBPM	–	–	–	≤0.063	0.5	≤0.063–0.5	–	–	–	0.5	1	≤0.063–2
PAPM	0.5	2	≤0.063–2	0.25	4	≤0.063–4	1	4	0.5–4	2	8	0.25–8
MEPM	≤0.063	0.25	≤0.063–0.5	≤0.063	0.25	≤0.063–0.25	0.125	0.25	≤0.063–0.25	0.25	0.5	≤0.063–1
DRPM	–	–	–	0.125	1	≤0.063–1	–	–	–	1	2	≤0.063–2
LVFX	≤0.063	≤0.063	≤0.063–≤0.063	≤0.063	≤0.063	≤0.063–≤0.063	≤0.063	0.25	≤0.063–0.25	≤0.063	≤0.063	≤0.063–≤0.063
TFLX	–	–	–	≤0.063	≤0.063	≤0.063–≤0.063	–	–	–	≤0.063	≤0.063	≤0.063–≤0.063

*ABPC* ampicillin, *CVA/AMPC* clavulanic acid/amoxicillin, *PIPC* piperacillin, *TAZ/PIPC* tazobactam/piperacillin, *CCL* cefaclor, *CDTR* cefditoren, *CFPN* cefcapene, *CPDX* cefpodoxime, *CFDN* cefdinir, *CFTM* cefteram, *CTM* cefotiam, *CTRX* ceftriaxone, *CTX* cefotaxime, *AZM* azithromycin, *CAM* clarithromycin, *RKM* rokitamycin, *TEL* telithromycin, *FRPM* faropenem, *TBPM* tebipenem, *PAPM* panipenem, *MEPM* meropenem, *DRPM* doripenem, *LVFX* levofloxacin, *TFLX* tosufloxacin

The MIC_90_ values of antimicrobial agents for *H. influenzae*, categorized by degrees of resistance, are as follows: in the BLNAS group, the oral antimicrobial agents with the lowest MIC_90_ were CDTR and LVFX (≤0.063 μg/ml) in phase 3, and LVFX and TFLX (≤0.063 μg/ml) in phase 4, whereas the intravenous agent with the lowest MIC_90_ was TAZ/PIPC (≤0.063 μg/ml) in both phase 3 and phase 4. In the BLNAI, BLNAR, and BLPAR groups, the oral antimicrobial agent with the lowest MIC_90_ was LVFX (≤0.063 μg/ml) in phase 3, and LVFX and TFLX (≤0.063 μg/ml) in phase 4. In the BLNAI group, the intravenous antimicrobial agent with the lowest MIC_90_ was TAZ/PIPC (0.125 μg/ml) in phase 3, and PIPC and TAZ/PIPC (0.125 μg/ml) in phase 4. In the BLNAR group, the intravenous antimicrobial agents with the lowest MIC_90_ were PIPC, TAZ/PIPC, and CTRX (0.25 μg/ml) in phase 3, and TAZ/PIPC (0.125 μg/ml) in phase 4. In the BLPAR group, the intravenous antimicrobial agent with the lowest MIC_90_ was TAZ/PIPC in both phase 3 and phase 4 (phase 3, ≤0.063 μg/ml; phase 4, 0.125 μg/ml). In the BLPACR group, the oral antimicrobial agent with the lowest MIC_90_ was CDTR and LVFX (0.25 μg/ml) in phase 3, and LVFX and TFLX (≤0.063 μg/ml) in phase 4, whereas the intravenous antimicrobial agent with the lowest MIC_90_ was TAZ/PIPC in both phase 3 and phase 4 (phase 3, 0.125 μg/ml; phase 4, ≤0.063 μg/ml).

The frequency of isolation was compared between drug-resistant strains and BLNAS strains in relationship to six background factors: sex, age, presence or absence of siblings, attendance or nonattendance at a daycare center, siblings’ attendance or nonattendance at a daycare center, and prior administration of antimicrobial agents within 1 month (Table 3). In phase 3, the isolation rate of drug-resistant strains was higher (*P* = 0.005) in cases with prior administration of antimicrobial agents.

**Table 3:** Number of cases of β-lactamase-nonproducing ABPC-sensitive strain (BLNAS) or β-lactamase-nonproducing ABPC-intermediately resistant strain (BLNAI) + β-lactamase-nonproducing ABPC-resistant strain (BLNAR) according to background factor

Background factor	Phase 3			Phase 4		
	Number of cases		Statistics	Number of cases		Statistics
	BLNAS	BLNAI + BLNAR		BLNAS	BLNAI + BLNAR	
Sex						
Boy	78	123	χ^2^	91	152	χ^2^
Girl	46	111	*P* = 0.0607	70	128	*P* = 0.6495
Age category						
Infant	26	40	χ^2^	28	74	χ^2^
Toddler	91	177	*P* = 0.4703	122	196	*P* = 0.0546
Schoolchild	13	17		11	11	
Sibling/siblings						
Yes	80	144	χ^2^	94	179	χ^2^
No	51	91	*P* = 0.9688	67	102	*P* = 0.2684
Group daycare						
Yes	78	141	χ^2^	88	147	χ^2^
No	48	83	*P* = 0.8467	70	108	*P* = 0.6972
Group daycare (siblings)						
Yes	62	114	χ^2^	70	122	χ^2^
No	8	16	*P* = 0.8552	21	37	*P* = 0.9722
Previous use of antimicrobial agents						
Yes	58	139	χ^2^	87	176	χ^2^
No	75	97	*P* = 0.0047	74	105	*P* = 0.0765
Penicillins	8	29		20	46	
Cephems	29	85		36	93	
Macrolides	21	55		40	74	
β-Lactam	36	103	χ^2^	53	123	χ^2^
Macrolides	21	55	*P* = 0.7832	40	74	*P* = 0.3754

*ABPC* ampicillin

## Discussion

The Drug-Resistant Pathogen Surveillance Group in Pediatric Infectious Disease has continued to conduct national surveillance for the antimicrobial susceptibility of *H. influenzae* since 2000. The previous study reported that the frequency of BLNAR strains dramatically increased from 28.8 % in phase 1 (2000–2001) to 59.3 % in phase 2 (2004) [3]. An ABPC MIC of 2 μg/ml or more was used as a criterion for the identification of BLNAR strains in phase 1 and phase 2. Had an ABPC MIC of 4 μg/ml or more been used as in the current study, the frequency of BLNAR strains would have decreased to 11.4 % and 43.4 % in phases 1 and 2, respectively. Nonetheless, the distribution of BLNAR strains still increased dramatically between phase 1 and phase 2.

In this study, the frequency of BLNAR strains was 38.3 % in phase 3 and 37.8 % in phase 4, indicating a downward tendency from its peak in phase 2. According to a report on the national surveillance conducted in Spain, the frequency of BLNAR strains gradually decreased from 13.5 % in 1996–1997 to 0.7 % in 2006–2007 [7]. Possible reasons for the decline in the number of BLNAR strains are changes in the number of prescriptions and clonal spread of sensitive strains. A positive correlation between the dosage of antimicrobial agents and the development of drug resistance has been observed with the use of population genetic methods [8]. Owing to the widespread use of guidelines in the pediatric field [9, 10], the proper use of antimicrobial agents may have been promoted in Japan.

In contrast to the decline in the number of BLNAR strains, the number of β-lactamase-producing strains, which had continued to decrease, from 8.3 % in phase 1 to 6.4 % in phase 2, and to 4.4 % in phase 3, increased to 8.7 % in phase 4. This difference was greatly influenced by the BLPACR strains, whose number increased from 1.6 % in phase 3 to 4.8 % in phase 4. The BLPACR strains, which have two mechanisms of antimicrobial resistance that comprise β-lactamase production and mutations in *ftsI* genes, show resistance to many β-lactams. High detection rates of BLPACR strains have been reported in France (13.9 %) from 1999 to 2000 [11] and in Spain (22.4 %) from 2005 to 2007 [12], and in some cases clonal dissemination of the BLPACR strains has been observed [12, 13]. A similar phenomenon may have happened in Japanese children; analysis of clonality using multilocus sequence typing or pulsed-field gel electrophoresis method will thus be required to explain the increase of BLPACR strains.

The BLNAR strains, which have mutations in the *ftsI* genes that make PBP3 with poor affinity for antimicrobial agents, showed reduced susceptibility to various types of β-lactams including cephems [2]. When the number of BLNAR strains rapidly increased between phase 1 and phase 2, the MIC_90_ values of most β-lactams showed a corresponding 2- to 4 fold increase [3]. On the other hand, the BLNAR strains showed a trend toward improved susceptibility after phase 2, when the number of BLNAR strains decreased and the MIC_90_ value of each β-lactam antimicrobial agent decreased from one half to one fourth between phase 2 and phase 3. There were no increases in MIC_90_ values of β-lactams between phase 3 and phase 4 with the exception of CDTR, CFPN, and PAPM, which showed a 2-fold increase. However, the MIC_90_ values of β-lactams in BLNAR strains were 2- to 4 fold higher in phase 3 and 2- to 8 fold higher in phase 4, when compared with BLNAS strains. As BLNAR strains show lower susceptibility to most oral β-lactams, it is considered that limited treatment options will continue to be a particular problem.

In this study, the antimicrobial agents with the lowest MIC_90_ were LVFX in phase 3 and LVFX and TFLX in phase 4. Fluoroquinolones, which are not affected by mutations in *ftsI* genes, showed lower MIC_50_ and MIC_90_ values of ≤0.063 μg/ml for the BLNAR strains. Although the MIC of LVFX was continuously measured in phases 3 and 4, no reduced susceptibility was observed. However, the emergence of fluoroquinolone-resistant *H. influenzae* strains has been detected in adult patients who use fluoroquinolones frequently [14]. Treatment with TFLX in pediatric patients with otitis media or pneumonia has been covered by insurance since 2009; the use of fluoroquinolones in pediatric patients can thus be expected to increase steadily. The proper use of fluoroquinolones is extremely important if we are to prevent fluoroquinolone-resistant strains from spreading in pediatric patients.

In Japan, Hib vaccine was introduced as a voluntary vaccination in December 2008, between phases 3 and 4. Two years after its introduction, at the end of 2010, publicly subsidized vaccines became available, and since then the incidence of invasive Hib infection has decreased because of an improvement in the vaccination rate [15]. In our study, 14 (3.6 %) and 23 Hib strains (4.8 %) were isolated in phase 3 and phase 4, respectively, indicating no decrease in the number of Hib cases after the introduction of the vaccine. However, the strains in phase 4 were acquired between January and June 2010, before the initiation of the publicly subsidized vaccine program; it is likely that the Hib vaccine was not then widely available. We will have to wait for the next phase of surveillance to confirm the effect of vaccination on Hib detection rates.

During phase 1 and phase 2 surveillance, the relationship between three background factors (age, prior administration of antimicrobial agents, and attendance at a daycare center) and the isolation rate of drug-resistant strains was examined, and there were significant differences in age and prior administration of antimicrobial agents in phase 2 [3]. In this study, in relationship to a total of six background factors, including the three factors from the previous study and three additional factors (sex, siblings, and siblings’ attendance at a daycare center), there were significant differences as regards prior administration of antimicrobial agents in phase 3.

In phase 3, the isolation rate of drug-resistant strains was high in those cases with prior administration of antimicrobial agents. In phase 2, there were differences related to the types of antimicrobial agents; the frequency of drug-resistant strains in the cases treated with β-lactams was higher than that in the cases treated with macrolides, while no differences were observed in phase 3. Although the mutations in PBP3 in the BLNAR strains are considered to be easily induced by the abuse of the oral cephems [2], no significant difference was found among penicillins, cephems, and macrolides. It is likely that each type of antimicrobial agent was prescribed in a balanced manner.

In relationship to other background factors, no significant difference was found in the frequency of drug-resistant strains. In phase 2, the isolation rate of drug-resistant strains was significantly higher in children under 3 years of age, while no such difference was found in phases 3 and 4 (data not shown). As there was also an apparent decrease in the number of the BLNAR strains in infants, similar future transitions deserve attention.

It has been 10 years since the establishment of this group. We would like to continue conducting surveillance so that we can provide useful information on the drug resistance of *H. influenzae* for use in clinical practice.

## References

[1] Hasegawa K, Chiba N, Kobayashi R, Murayama SY, Iwata S, Sunakawa K (2004). Rapidly increasing prevalence of β-lactamase-nonproducing, ampicillin-resistant *Haemophilus influenzae* type b in patients with meningitis. Antimicrob Agents Chemother.

[2] Hasegawa K, Kobayashi R, Takada E, Ono A, Chiba N, Morozumi M, et al. High prevalence of type b β-lactamase-nonproducing ampicillin-resistant *Haemophilus influenzae* in meningitis: the situation in Japan where Hib vaccine has not been introduced. J Antimicrob Chemother 2006;57:1077–1082. 10.1093/jac/dkl142 16617062

[3] Sakata H, Toyonaga Y, Sato Y, Hanaki H, Nonoyama M, Oishi T (2009). Nationwide survey of the development of drug-resistance in the pediatric field: drug sensitivity of *Haemophilus influenzae* in Japan. J Infect Chemother.

[4] Clinical and Laboratory Standards Institute (2006). Methods for dilution antimicrobial susceptibility tests for bacteria that grow aerobically; approved standard. Seventh edition M7–A7.

[5] Clinical and Laboratory Standards Institute. Performance standards for antimicrobial susceptibility testing; 17th informational supplement. M100-S17. Wayne, PA: Clinical and Laboratory Standards Institute; 2007.

[6] Falla TJ, Crook DW, Brophy LN, Maskell D, Kroll JS, Moxon ER (1994). PCR for capsular typing of *Haemophilus influenzae*. J Clin Microbiol.

[7] Pérez-Trallero E, Martín-Herrero JE, Mazón A, García-Delafuente C, Robles P, Iriarte V (2010). Antimicrobial resistance among respiratory pathogens in Spain; latest data and change over 11 years (1996–1997 to 2006–2007). Antimicrob Agents Chemother.

[8] Austin DJ, Kristinsson KG, Anderson RM (1999). The relationship between volume of antimicrobial consumption in human communities and the frequency of resistance. Proc Natl Acad Sci USA.

[9] The Committee for Guidelines for the Management of Respiratory Infectious Diseases in Children in Japan (2011) Guidelines for the management of respiratory infectious diseases in children in Japan (in Japanese). Japanese Society of Pediatric Pulmonology, Japanese Society for Pediatric Infectious Diseases, Tokyo

[10] The Subcommittee on Clinical Practice Guidelines for the Diagnosis and Management of Acute Otitis Media in Children (2009) Clinical practice guideline for diagnosis and management of acute otitis media (AOM) in children in Japan (in Japanese). Japan Otological Society, Japan Society for Pediatric Otorhinolaryngology, Japan Society for Infectious Diseases in Otolaryngology, Tokyo

[11] Dabernat H, Delmas C, Seguy M (2002). Diversity of β-lactam resistance-conferring amino acid substitutions in penicillin-binding protein 3 of *Haemophilus influenzae*. Antimicrob Agents Chemother.

[12] Sevillano D, Giménez MJ, Cercenado E, Cafini F, Gené A, Alou L (2009). Genotypic versus phenotypic characterization, with respect to β-lactam susceptibility, of *Haemophilus influenzae* isolates exhibiting decreased susceptibility to β-lactam resistance markers. Antimicrob Agents Chemother.

[13] Barbosa AR, Giufré M, Cerquetti M, Bajanca-Lavado MP (2011). Polymorphism in *ftsI* gene and β-lactam susceptibility in Portuguese *Haemophilus influenzae* strains: clonal dissemination of β-lactamase-positive isolates with decreased susceptibility to amoxicillin/clavulanic acid. J Antimicrob Chemother.

[14] Yokota S, Ohkoshi Y, Sato K, Fujii N (2008). Emergence of fluoroquinolone-resistant *Haemophilus influenzae* strains among elderly patients but not among children. J Clin Microbiol.

[15] Ubukata K, Chiba N, Morozumi M, Iwata S, Sunakawa K. Longitudinal surveillance of *Haemophilus influenzae* isolates from pediatric patients with meningitis throughout Japan, 2000–2011. J Infect Chemother. doi: 10.1007/s10156-012-0448-x. 10.1007/s10156-012-0448-x 22806445

